# Glucose Management during Insulinoma Resection Using Real-Time Subcutaneous Continuous Glucose Monitoring

**DOI:** 10.1155/2018/6248467

**Published:** 2018-06-07

**Authors:** Yuki Sugiyama, Chiaki Kiuchi, Maiko Suzuki, Yuki Maruyama, Ryo Wakabayashi, Yasunari Ohno, Shugo Takahata, Takumi Shibazaki, Mikito Kawamata

**Affiliations:** ^1^Department of Anesthesiology and Resuscitology, Shinshu University School of Medicine, Japan; ^2^Division of Pediatric Surgery, Department of Surgery, Shinshu University School of Medicine, Japan; ^3^Department of Pediatrics, Shinshu University School of Medicine, Japan

## Abstract

Insulinoma is a rare neuroendocrine tumor that causes hypoglycemia due to unregulated insulin secretion. Blood glucose management during insulinoma resection is therefore challenging. We present a case in which real-time subcutaneous continuous glucose monitoring (SCGM) in combination with intermittent blood glucose measurement was used for glycemic control during surgery for insulinoma resection. The SCGM system showed the trends and peak of interstitial glucose in response to glucose loading and the change of interstitial glucose before and after insulinoma resection. These data were helpful for adjusting the glucose infusion; therefore, we think that an SCGM system as a supportive device for glucose monitoring may be useful for glucose management during surgery.

## 1. Introduction

Insulinoma is a rare neuroendocrine tumor that causes hypoglycemia, and surgical resection is indicated for all localized tumors [1]. Ten percent of insulinomas occur as a part of multiple endocrine neoplasia type 1 (MEN-1), in which insulinoma occurs at a relatively young age [2]. It is challenging for anesthesiologists to control blood glucose (BG) during surgery for insulinoma resection [3]. A subcutaneous continuous glucose monitoring (SCGM) system, which allows real-time monitoring of interstitial glucose (IG), has been developed and is used in diabetic patients [4]. We present a case of insulinoma in which an SCGM system provided the change of IG and played a role as a supportive device for glucose management during insulinoma resection.

Written informed consent was obtained from the patient's parents for publication of this case report.

## 2. Case Presentation

A 12-year-old girl (height, 144 cm; weight, 40 kg) was presented to the hospital with an episode of seizure with impaired consciousness. Her BG at that time was 60 mg/dL (normal value of casual BG: 70–200 mg/dL). Glucose was administered and she recovered consciousness. She had no significant comorbidities prior to hospital admission. From her family history and further investigation, she was diagnosed as having insulinoma and hyperparathyroidism in MEN-1. Arterial phase images of computed tomography showed a vascularity-rich tumor of 20 mm in diameter located in the head of the pancreas. The feeding artery of the tumor was not clearly demonstrated by angiography and selective arterial calcium injection. We considered that extended surgical procedures such as pancreatoduodenectomy might be required depending on intraoperative findings; therefore, open abdominal surgery rather than laparoscopic surgery was scheduled. Her intact parathyroid hormone level was 66.0 pg/ml (normal value: 10–65 pg/mL) and her adjusted serum calcium level was 10.3 mg/dL (normal value: 8.7–9.9 mg/dL), although parathyroid ultrasound examination revealed no parathyroid tumor. Other tumors complicated with MEN-1 were not detected. The results of other preoperative examinations were unremarkable. On the day before surgery, an Enlite™ sensor of MiniMed™ 620G (Medtronic Diabetes, Northridge, CA, USA) SCGM system was inserted into her upper arm. Although MiniMed 620G was combined with an insulin pump, we did not use the pump. The SCGM system was calibrated as recommended by the manufacturer with capillary BG measured by OneTouch® UltraVue™ Blood Glucose Meter (Johnson & Johnson, New Brunswick, NJ, USA).

**Figure 1:**
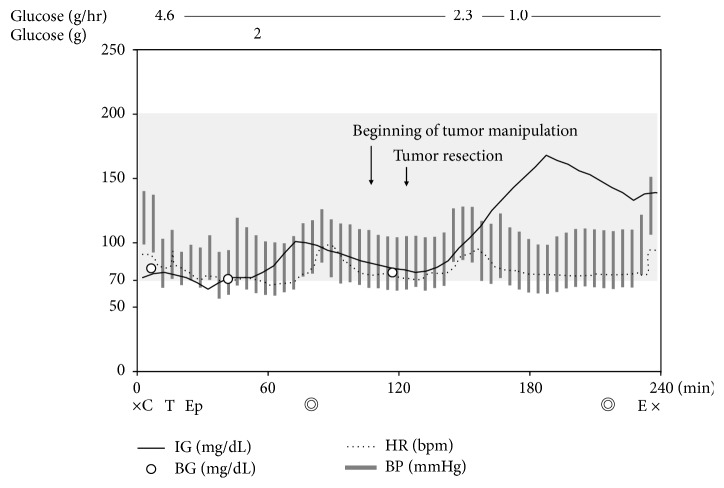
Changes of interstitial glucose level measured by the subcutaneous continuous glucose monitoring system (IG) (black line), blood glucose level (BG) (white circle), and hemodynamics during general anesthesia for resection of insulinoma. The light grey zone indicates the normal value of casual BG (70–200 mg/dL). The dotted line indicates heart rate (HR) and the grey bar indicates blood pressure (BP). C indicates calibration of the subcutaneous continuous glucose monitoring system; T: tracheal intubation; Ep: epidural catheterization; E: extubation; *◎*: start or end of the operation; ×: start or end of anesthesia.

No premedication was given and she walked into the operating room. Capillary BG was 80 mg/dL and the SCGM system was calibrated. Continuous glucose infusion was started at 4.6 g/hr, and general anesthesia was induced with 80 mg of propofol, 50 mcg of fentanyl, and 0.02 mcg/kg/min of remifentanil. Muscle relaxation was obtained by administration of 30 mg of rocuronium, and the trachea was intubated. After induction of general anesthesia, an epidural catheter was inserted at the eighth and ninth thoracic interspace. Anesthesia was maintained with 1.7% sevoflurane, 0.01–0.02 mcg/kg/min of remifentanil, and intermittent thoracic epidural administration of 0.25% levobupivacaine (3 ml). An arterial catheter was inserted, and arterial BG measured by an ABL800 Flex Blood Gas Analyzer (Radiometer, Brea, CA, USA) was 71 mg/dL, which was compatible with IG of 73 mg/dL. An additional 2 g of glucose was given intravenously before the start of surgery, and IG increased from 77 mg/dL to 101 mg/dL at 20 minutes (Figure 1). After that, IG mildly decreased and returned to almost the same level after 1 hour. The surgeon informed us that the tumor would be removed by enucleation shortly. Just before tumor resection, arterial BG was 76 mg/dL and IG was 80 mg/dL. At 20 minutes after tumor resection, IG showed a rapid increase. We therefore decreased the continuous glucose infusion rate to the usual dose of 1 g/hr, and IG gradually decreased and was stabilized at about 140 mg/dL. During surgery, blood pressure was between 90/50 and 120/70 mmHg, and heart rate was between 60 and 90 bpm. She was extubated in the operating room and transferred to the general surgery ward. The operation time was 2 h and 24 min, and the anesthesia time was 3 h and 53 min. Postoperative pain was controlled well by continuous epidural analgesia (12 mcg of fentanyl and 0.2% levobupivacaine at 4 ml/hr) and administration of 600 mg of acetaminophen (every 6 hr). Her postoperative course was uneventful and she was discharged on POD 10.

## 3. Discussion

An SCGM system has been used to monitor glucose level and has improved long-term glycemic control in diabetic patients [5]. The MiniMed 620G SCGM system is composed of a small sensor (6 g) and a transmission device (130 g), which can record real-time data from its wireless sensor. The SCGM sensor, which has a small electrode inserted into subcutaneous tissue, can measure IG by the glucose oxidase method.

In this case, we used the MiniMed 620G SCGM system as a supportive device for continuous monitoring of IG trends in combination with intermittent BG measurements. Fortunately, the operation was completed within 2.5 hr and resection of the insulinoma per se was completed within 30 min. During surgery, we performed 3 measurements of BG and those measurements showed close correlation with IG measurements (Figure 1). A single injection of glucose was administered before the surgery because the patient's BG remained relatively low despite continuous glucose infusion. IG gradually increased in response to the single glucose injection and the delay of IG to BG in our case was found to be about 20 min from this response (Figure 1). Based on this finding, we could estimate the change in BG by the real-time change in IG. A gradual decrease and increase in IG were also seen before and after resection of the insulinoma, respectively (Figure 1). Since the SCGM system provided trends and peak of IG change, which are difficult to know by only BG measurement, SCGM could be considered as being complementary to intermittent BG measurement.

The SCGM system can provide such useful information for glucose management; however, attention should be paid to the fact that the SCGM system has not been completely established as a substitutable device for BG measurement in the perioperative period because dissociation between BG and IG sometimes occurs [6–9]. For this reason, BG measurement is recommended before administration of insulin and/or glucose, and we avoided the use of automatic control by the insulin pump of the SCGM system. The dissociation is considered to be caused by some factors including the delay of IG to BG as shown in our case. The delay of IG to BG in diabetic patients has been reported to range from 4 to 10 minutes in the abdomen [10] and to be 12 minutes in both the abdomen and arm [11]. Other possible factors related to sensor performance are the site of sensor insertion, physiological changes including impaired peripheral perfusion induced by hypotension and/or use of vasopressors [6, 7], and calibration. The site for sensor insertion recommended by the manufacturer is a site where subcutaneous fat is thick, such as the upper arm, belly, upper hip, and thigh. Involvement of peripheral perfusion in sensor performance was indicated in some studies [6, 7]; however, another study demonstrated that the accuracy of SCGM was not influenced by the change of peripheral perfusion [12]. The timing and interval of calibration are also important factors to accurately use the SCGM system, and it has been shown that enhanced calibrations improved sensor accuracy [13]. Since such many factors have the potential to change the sensor accuracy, additional calibrations are recommended when the physiological situation drastically changes or surgery is prolonged and extended.

The usefulness of continuous glucose monitoring during insulinoma resection using an artificial endocrine pancreas has been reported [14]. Although an artificial endocrine pancreas can strictly control BG by automatically measuring BG and administering both glucose and insulin, it requires a large and complicated machine (36 kg) with additional venous catheterization. On the other hand, the SCGM system is small, less invasive, and easy to use. SCGM might be more acceptable to anesthesiologists and patients in clinical use.

In conclusion, we showed the utility of the use of a combination of SCGM and intermittent BG measurement for glucose management during insulinoma resection.
